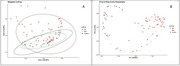# Gut Microbiome Alterations Across Cognitive Decline in Uganda: Phylogenetic and Compositional Insights

**DOI:** 10.1002/alz70855_097665

**Published:** 2025-12-23

**Authors:** Kamada Lwere

**Affiliations:** ^1^ Makerere University, Kampala, Uganda, Uganda

## Abstract

**Background:**

Alzheimer's disease (AD) is the leading cause of global cognitive decline. However, its mechanisms remain poorly understood in sub‐Saharan Africa (SSA), where genetic, dietary, and environmental factors differ significantly. Emerging evidence links the gut microbiota to AD through neuroinflammation and gut‐brain axis dysfunction. This study investigated phylogenetic and compositional microbiome shifts in AD, Mild Cognitive Impairment (MCI), and healthy controls in Uganda, providing novel insights into microbial dysbiosis and its role in cognitive decline in low‐resource settings.

**Method:**

In this cross‐sectional study, stool samples from 104 participants (AD: 77; MCI: 14; controls: 13) were analyzed using 16S rRNA sequencing (V3–V4 region), with DADA2 generating amplicon sequence variants (ASVs). Beta diversity was assessed using Weighted UniFrac (phylogenetic differences) and Bray‐Curtis (compositional differences) metrics. Principal Coordinate Analysis (PCoA) was used to visualize clustering patterns, whereas group differences were assessed using Permutational Multivariate Analysis of Variance (PERMANOVA) at *p* < 0.05.

**Result:**

Beta diversity analysis revealed distinct microbial shifts that were linked to cognitive decline. Weighted UniFrac PCoA showed clear clustering, with Axis 1 (39.46% variation) separating AD patients from controls and Axis 2 (16.28%) capturing within‐group variability, particularly in AD. The MCI group occupied an intermediate position, reflecting the microbial gradient associated with cognitive decline. Confidence ellipses highlighted minimal overlap between AD and controls, whereas MCI partially overlapped with both groups, suggesting a transitional profile. Bray‐Curtis PCoA confirmed compositional differences, with PC1 (28.66%) separating AD from controls and PC2 (14.44%) capturing MCI dispersion. PERMANOVA confirmed significant group‐level differences (Weighted UniFrac: R² = 0.18, *p* = 0.001; Bray‐Curtis: R² = 0.21, *p* = 0.001), with the strongest divergence between AD and controls (*p* = 0.001), and significant differences between AD and MCI (*p* = 0.005).

**Conclusion:**

Distinct microbial shifts across AD, MCI, and control groups highlight the role of the gut microbiome in neurodegeneration. The transitional profile of MCI underscores its potential as an early marker of dysbiosis, supporting the development of microbiome‐targeted strategies for the early detection and intervention of Alzheimer's disease.